# Clinical outcomes of cement gun–assisted intramedullary nailing for femoral bone metastases: a retrospective case series

**DOI:** 10.1186/s12891-026-10109-3

**Published:** 2026-06-18

**Authors:** Sei Morinaga, Katsuhiro Hayashi, Shinji Miwa, Takashi Higuchi, Hirotaka Yonezawa, Yohei Asano, Satoru Demura

**Affiliations:** Department of Orthopaedic Surgery, Graduate School of Medical Sciences, Kanazawa University, 13-1 Takara-Machi, Kanazawa, 920-8640 Ishikawa Japan

**Keywords:** Bone metastasis, Femur, Intramedullary nailing, Cement gun, Cement augmentation, Pathological fracture

## Abstract

**Background:**

Surgical stabilization for femoral bone metastases is commonly performed to prevent or treat pathological fractures; however, local tumor progression may result in implant failure and reoperation. Cement gun–assisted intramedullary nailing (CG-IMN) allows extensive polymethylmethacrylate (PMMA) filling within the medullary canal, providing enhanced mechanical stability and facilitating early weight-bearing. This study aimed to evaluate the clinical outcomes of CG-IMN for femoral bone metastases.

**Methods:**

We retrospectively reviewed 11 consecutive patients who underwent CG-IMN for femoral bone metastases at our institution between January 2008 and March 2025. Clinical data included patient demographics, primary tumor histology, presence of pathological fracture, revised Katagiri score, performance status (PS), lesion location, operative data, complications, reoperation rate, and ambulatory status at 1 month after surgery.

**Results:**

The mean age was 72.5 years. Seven patients had impending fractures and four had pathological fractures. The mean revised Katagiri score was 5.3. PS improved significantly from 3.5 preoperatively to 1.4 postoperatively (*p* < 0.01). Lesions involved the proximal femur (*n* = 3), diaphysis (*n* = 5), or both (*n* = 3). Mean blood loss was 319 mL and operative time was 191 min. At 1 month, 9 of 11 patients (81.8%) were ambulatory. One patient (9.1%) required reoperation due to implant breakage after a fall. The 1-year implant survival rate was 90%.

**Conclusions:**

CG-IMN provides stable fixation and functional improvement and may represent a useful surgical option, although further studies are required.

## Introduction

Bone metastases represent the most common malignant tumors of the skeletal system and represent a substantial clinical burden worldwide, with approximately 350,000 deaths annually in the United States associated with metastatic bone disease [1]. The femur is one of the most frequently affected long bones, and metastatic involvement often causes pain, functional impairment, and pathological fractures, leading to a marked reduction in quality of life [2].

Surgical stabilization is commonly required to restore skeletal stability, relieve pain, and allow early mobilization in patients with femoral metastases [3]. Intramedullary nailing (IMN) is widely used for the treatment of metastatic femoral lesions because it provides effective mechanical stabilization through a relatively minimally invasive procedure and allows early weight-bearing [4]. In oncologic patients with limited life expectancy, less invasive procedures that provide sufficient stability during the expected survival period are generally preferred.

However, implant failure remains an important concern after IMN for metastatic femoral lesions. Residual tumor within the bone may continue to grow despite systemic therapy or radiotherapy, leading to progressive osteolysis and mechanical weakening of the surrounding bone [5]. Jian W et al. have reported implant failure rates of approximately 14% following intramedullary fixation for metastatic femoral fractures due to tumor progression and inadequate mechanical support [6]. Implant breakage and the need for revision surgery have been associated with longer patient survival and insufficient structural reinforcement around the implant [7].

Polymethylmethacrylate (PMMA) bone cement has been widely used in orthopedic oncology to improve mechanical stability and reduce tumor-related complications [8]. PMMA injection provides structural reinforcement of osteolytic bone lesions and can significantly relieve pain and decrease the risk of secondary fractures [9]. Cement augmentation combined with intramedullary fixation may therefore enhance mechanical durability and reduce the risk of implant failure in metastatic femoral lesions. However, conventional manual cement injection may result in incomplete or uneven filling of the medullary canal, particularly in long osteolytic lesions. The use of a cement gun allows extensive filling of the medullary canal with PMMA, potentially providing more uniform mechanical support along the entire length of the nail. In addition, extensive cement filling may reduce the potential space for tumor progression and further structural weakening of the bone. Despite these theoretical advantages, clinical evidence regarding the outcomes and durability of cement gun–assisted intramedullary nailing (CG-IMN) for femoral bone metastases remains limited.

The purpose of this study was to evaluate the clinical outcomes, functional improvement, and implant durability of CG-IMN in patients with femoral bone metastases treated at our institution.

## Materials and methods

### Study design and patients

We retrospectively reviewed 11 consecutive patients who underwent CG-IMN for femoral bone metastases at our institution between January 2008 and March 2025. Patients were followed until death or the last clinical follow-up. The median follow-up period was 12 months (range, 1–83 months). Clinical data collected included patient demographics, primary tumor histology, presence of pathological fracture, preoperative revised Katagiri score, pre- and postoperative performance status (PS), lesion location, intraoperative blood loss, operative time, reoperation rate, cement-related complications, and ambulatory status at 1 month after surgery. Performance status was evaluated using the Eastern Cooperative Oncology Group (ECOG) Performance Status scale [10]. The revised Katagiri score, a prognostic scoring system for patients with skeletal metastases, and ECOG performance status were retrospectively assessed by the first author using predefined criteria based on electronic medical records. The revised Katagiri score [11] was assessed preoperatively because it is intended to estimate survival and assist treatment decision-making in patients with skeletal metastases.

### Indications for CG-IMN

The indication for CG-IMN in this study was the presence of metastatic femoral lesions with either impending or established pathological fractures in patients with multiple metastases. Surgical stabilization was considered when the expected prognosis was estimated to be more than several months but less than one year, based on the overall clinical condition and oncological status.

### Surgical procedure

**Fig. 1:**
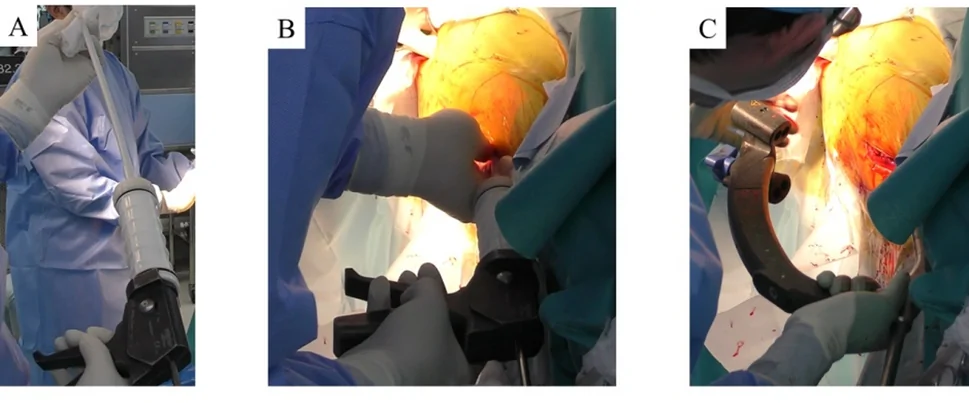
Surgical procedure of cement gun–assisted intramedullary nailing. **A** Preparation of a cement gun equipped with a long, narrow humeral cement nozzle (Stryker; 9.5 mm in diameter and 354 mm in length). **B** Insertion of the cement gun through the proximal femoral entry point for intramedullary nailing, followed by cement injection into the femoral canal. **C** Insertion of the intramedullary nail after filling the medullary canal with polymethylmethacrylate

**Fig. 2:**
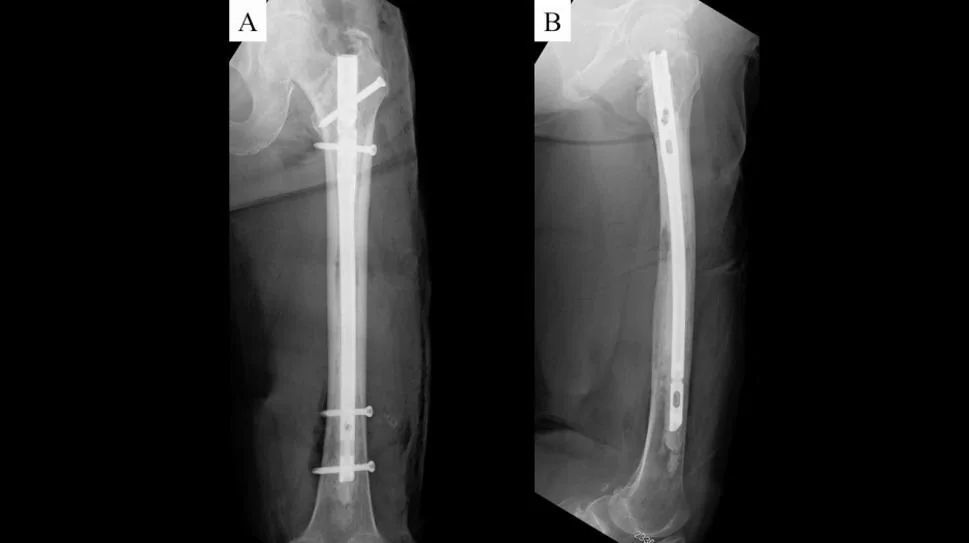
Postoperative radiographs after cement gun–assisted intramedullary nailing (CG-IMN). **A** Representative anteroposterior radiograph of the femur after CG-IMN. **B** Representative lateral radiograph of the femur after CG-IMN

All procedures were performed with the patient positioned on a traction table under fluoroscopic guidance. The planned nail length and the insertion angle of the femoral neck screw were confirmed preoperatively under fluoroscopy. PMMA cement (Simplex P Bone Cement; Stryker, Mahwah, NJ, USA) was prepared and loaded into a cement gun system. The femoral canal was over-reamed by approximately 2 mm relative to the nail diameter. Cement was injected when it reached a doughy consistency and no longer adhered to surgical gloves. Although no specific measures were used to prevent fat embolism or cement embolism, close communication with the anesthesiology team was maintained during cement injection. A long, narrow humeral cement nozzle (Stryker; diameter 9.5 mm, length 354 mm) was inserted into the femoral intramedullary canal, and cement injection was performed under fluoroscopic guidance (Fig. 1). Cement was injected from the distal portion of the canal toward the proximal direction to achieve extensive filling of the intramedullary space. After confirming adequate cement filling, the intramedullary nail was inserted into the femoral canal under fluoroscopic guidance. The alignment and depth of the nail were verified according to the preoperative plan, and proximal and distal interlocking screws were inserted to complete fixation (Fig. 2).

### Statistical analysis

All statistical analyses were performed using EZR (Saitama Medical Center, Jichi Medical University, Saitama, Japan) [12], which is a graphical user interface for R. Changes in PS before and after surgery were analyzed using the Wilcoxon signed-rank test. Implant survival was evaluated using the Kaplan–Meier method. Implant failure was defined as reoperation required because of mechanical failure at the metastatic lesion site. Patients who died without implant failure or were alive at the last follow-up were censored. A *p*-value < 0.05 was considered statistically significant.

### Ethical approval

This study was approved by the institutional review board of our institution, and the requirement for informed consent was waived due to the retrospective nature of the study.

## Results

A total of 11 patients (7 men and 4 women) underwent CG-IMN for femoral bone metastases. The primary tumor histologies included renal cell carcinoma (*n* = 3), lung cancer (*n* = 2), vaginal cancer (*n* = 1), laryngeal squamous cell carcinoma (*n* = 1), hepatocellular carcinoma (*n* = 1), breast cancer (*n* = 1), neuroendocrine carcinoma (*n* = 1), and prostate cancer (*n* = 1).

The mean age was 72.5 years (range, 63–82). Seven patients presented with impending fractures and four with established pathological fractures. The mean preoperative revised Katagiri score was 5.3 (range, 2–8). Performance status significantly improved from 3.5 (range, 3–4) preoperatively to 1.4 (range, 0–3) postoperatively (*p* < 0.01, Wilcoxon signed-rank test).

Lesions involved the proximal femur (*n* = 3), diaphysis (*n* = 5), or both regions (*n* = 3). Femoral neck screws were inserted in six patients according to lesion location and implant design. The mean intraoperative blood loss was 319 mL (range, 10–960), and the mean operative time was 191.2 min (range, 101–418). Intraoperative blood transfusion was required in six patients (three patients received 2 units of red blood cells (RBCs), two patients received 4 units of RBCs, and one patient received 4 units of RBCs plus 8 units of fresh frozen plasma), whereas no patient required postoperative transfusion. Cement leakage from the metastatic lesion was observed in six patients; however, no patient developed clinical symptoms related to cement leakage, and no additional surgical intervention was required. No patients received radiotherapy before surgery.

**Fig. 3:**
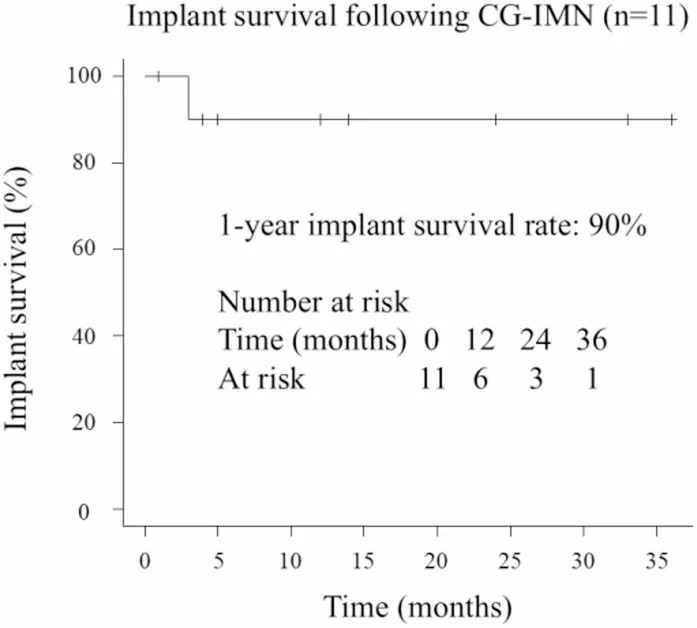
Kaplan–Meier curve of implant survival after cement gun–assisted intramedullary nailing (CG-IMN). The 1-year implant survival rate was 90%. Implant failure was defined as reoperation due to mechanical failure

At 1 month after surgery, 9 of 11 patients (81.8%) were ambulatory, including one walking independently, three with a cane, two with crutches, and three with a walker. At 3 months postoperatively, two patients had died, two were lost to follow-up after transfer to another institution, one remained wheelchair-dependent, one ambulated with crutches, two ambulated with a cane, two walked independently, and one underwent revision surgery. At 1 year postoperatively, four patients had died, two were lost to follow-up, one remained wheelchair-dependent, one ambulated with crutches, one ambulated with a cane, one walked independently, and one had undergone revision surgery. Among the three patients who required walker-assisted ambulation at 1 month postoperatively, two subsequently died, whereas one improved to cane-assisted ambulation. One patient (9.1%) required reoperation at 3 months due to implant breakage after a fall, which occurred at an empty screw hole rather than as a result of tumor progression or mechanical insufficiency of the construct (Table 1). Removal of the distal implant was technically challenging because of cement augmentation, and reconstruction was subsequently performed using a proximal femoral replacement. The 1-year implant survival rate was 90% (Fig. 3). Apart from one case of transient intraoperative hypotension and oxygen desaturation and one revision surgery for implant breakage after a fall, no other perioperative complications were observed.

**Table 1:** Patient characteristics and clinical outcomes of patients who underwent cement gun–assisted intramedullary nailing for femoral bone metastases

Characteristic		Number
Sex	Male	7
	Female	4
Mean age (years old)		72.5 (63–82)
Primary tumor	Renal cell carcinoma	3
	Lung cancer	2
	Vaginal cancer	1
	Laryngeal squamous cell carcinoma	1
	Hepatocellular carcinoma	1
	Breast cancer	1
	Neuroendocrine carcinoma	1
	Prostate cancer	1
Impending or pathological fracture	Impending	7
	Pathological	4
Lesion location	Proximal	3
	Diaphysis	5
	Both	3
Mean revised Katagiri score		5.3 (2–8)
Performance status	Pre	3.5 (3–4)
	Post	1.4 (0–3)
Mean intraoperative blood loss (ml)		319 (10–960)
Mean operative time (minutes)		191.2 (101–418)
Ambulation status at 1 month after surgery	Independently	1
	Cane	3
	Crutches	2
	walker	3
	Wheelchair	2
Reoperation	-	10
	+	1

One patient experienced transient intraoperative hypotension and oxygen desaturation associated with cement use, with no persistent complications.

### Discussion

Bone metastases of the long bones frequently result in severe pain, structural weakening of the bone, and pathological fractures, leading to marked deterioration in patient mobility and quality of life [13]. Surgical stabilization is therefore commonly required to restore mechanical stability and maintain activities of daily living in patients with advanced malignancies. Previous studies have shown that surgical treatment of femoral metastases can provide rapid pain relief and allow early mobilization and weight-bearing, which are important goals in patients with limited life expectancy [2, 14].

IMN is widely used for the treatment of metastatic lesions of long bones because it protects a long segment of bone, requires relatively limited soft tissue dissection, and preserves periosteal blood supply while providing stable fixation through proximal and distal interlocking screws [15, 16]. These advantages make IMN particularly suitable for patients with metastatic disease, in whom minimally invasive stabilization and early postoperative recovery are desirable. However, IMN without cement augmentation may be susceptible to mechanical failure over time, particularly in osteolytic metastatic lesions [6]. IMN functions primarily as a load-sharing device rather than a load-bearing construct; therefore, progressive tumor-related bone destruction can lead to implant fatigue and eventual failure.

To address this limitation, PMMA bone cement has been used as an adjunct to intramedullary fixation to improve mechanical stability [17]. PMMA cement serves as a bone substitute with high mechanical strength and the ability to fill defects caused by tumor destruction or surgical curettage [18]. Cement augmentation can reinforce residual bone, improve fixation of the nail along its entire length rather than only at the locking screw sites, and enhance rotational stability of the construct. In addition to its mechanical effects, PMMA undergoes an exothermic polymerization reaction during curing, which may produce a local thermonecrotic effect on tumor cells [19, 20]. These biomechanical and biological properties may contribute to improved implant stability and reduced risk of mechanical failure.

Previous studies have described IMN combined with percutaneous cement augmentation using vertebroplasty needles or manual syringe injection [21, 22]. While these techniques may improve fixation, they often provide limited control over the distribution of cement within the intramedullary canal. However, such systems require specially designed implants and may not be widely available. In contrast, the technique used in the present study allows controlled intramedullary cement injection using a cement gun and a long intramedullary nozzle, enabling extensive cement filling of the femoral canal without the need for specialized implants. This approach allows uniform cement augmentation along the entire length of the nail and may therefore provide improved mechanical support.

In the present study, CG-IMN resulted in improvement in PS and a high rate of postoperative ambulation. At one month after surgery, more than 80% of patients were able to ambulate, indicating that this technique provided sufficient mechanical stability to allow early functional recovery. Early restoration of mobility is particularly important in patients with metastatic disease, as the main goal of surgery is to improve quality of life and maintain independence during the remaining lifespan [3].

Another important finding of this study was the relatively low rate of implant failure. Only one patient required reoperation, resulting in a reoperation rate of 9.1% and a one-year implant survival rate of 90%. This failure occurred after a traumatic fall and at an empty screw hole rather than at the metastatic lesion itself, suggesting that the construct provided sufficient mechanical stability at the tumor site. These findings support the hypothesis that extensive intramedullary cement augmentation using a cement gun can enhance the mechanical durability of intramedullary fixation even in the presence of progressive metastatic bone destruction.

Previous studies have reported relatively high reoperation rates after surgical treatment of femoral bone metastases. For example, reoperation rates of approximately 16–21% have been reported in patients undergoing fixation for metastatic lesions of the proximal femur [23, 24]. Compared with these reports, the reoperation rate observed in the present study was relatively low. Although direct comparisons are limited by differences in patient populations and surgical techniques, the extensive cement augmentation achieved using the cement gun technique may have contributed to improved mechanical stability and reduced implant failure.

This study has several limitations. First, it was a retrospective analysis conducted at a single institution with a relatively small number of patients. Because of the limited sample size, subgroup analysis according to lesion location (proximal femur versus diaphyseal lesions) was not performed. Second, the follow-up period was limited, reflecting the poor prognosis of many patients with metastatic disease. Third, the absence of a control group treated with conventional intramedullary nailing without cement augmentation makes it difficult to directly evaluate the comparative effectiveness of this technique.

CG-IMN may be particularly suitable for patients with multiple metastatic lesions and limited life expectancy requiring durable fixation and early mobilization. In contrast, patients with potentially curable solitary lesions may be better treated with oncological resection and reconstruction. In patients with severely compromised general condition, intramedullary nailing without cement augmentation may be preferred to reduce operative invasiveness. Nevertheless, the present study provides preliminary clinical evidence supporting the feasibility of cement gun–assisted intramedullary cement augmentation in the surgical treatment of femoral bone metastases.

Although this study was limited by its retrospective design and small sample size, cement gun–assisted intramedullary nailing was associated with a low complication rate and a low rate of implant-related reoperation in this series. These findings suggest that extensive cement augmentation may improve implant durability in selected patients with femoral bone metastases. Larger comparative studies are required to confirm these findings.

## Data Availability

The data generated or analyzed during this study are included in this published article. Additional data are available from the corresponding author on reasonable request.

## Abbreviations

CG-IMN: Cement gun–assisted intramedullary nailing
PMMA: Polymethylmethacrylate
PS: Performance status
RBCs: Red blood cells
